# Breast Cancer Survivors’ Lived Experience of Adjuvant Hormone Therapy: A Thematic Analysis of Medication Side Effects and Their Impact on Adherence

**DOI:** 10.3389/fpsyg.2022.861198

**Published:** 2022-05-06

**Authors:** Maryam Ibrar, Nicola Peddie, Sommer Agnew, Amanda Diserholt, Leanne Fleming

**Affiliations:** ^1^School of Psychological Sciences and Health, University of Strathclyde, Glasgow, United Kingdom; ^2^School of Psychology and Life Sciences, Canterbury Christ Church University, Canterbury, United Kingdom

**Keywords:** breast cancer, hormone therapy, lived experience, side effects, adherence

## Abstract

**Objectives:**

Side effects from Hormone Therapy (HT) impact medication adherence in breast cancer survivors. Understanding the most distressing HT side effects and their impacts may inform the development and implementation of interventions to alleviate these side effects and maximise HT adherence. This study aimed to explore the lived experience of adjuvant HT and understand the impact of HT side effects on adherence in a sample of breast cancer survivors.

**Methods:**

Twenty-five female breast cancer survivors who were currently taking adjuvant HT participated in the study. One-to-one, online, semi-structured interviews were conducted to explore (i) specific side effects from HT and (ii) the impact of these side effects on HT adherence. Data were analysed using Thematic Analysis.

**Results:**

The most commonly reported side effects were sleep disturbance, hot flashes, anxiety, and joint pain. Data exploring the impacts of these side effects on HT adherence were thematically synthesised into four analytical themes: “A bitter pill to swallow,” “Seeking relief,” “Taking control,” and “The only way out is through.” These themes encompass 14 sub-themes that encapsulate participants’ daily struggle with HT side effects and the coping strategies developed to manage these.

**Conclusion:**

Adverse side effects from HT, such as sleep disturbance, hot flashes, pain and anxiety, impair quality of life and increase the likelihood of participants’ becoming non-adherent to HT medication. In order to maximise HT adherence and reduce breast cancer mortality, HT side effects should be closely monitored to identify individuals who would benefit from targetted intervention strategies aimed at alleviating these side effects.

## Introduction

Breast cancer is the most prevalent cancer in the United Kingdom, accounting for 15% of all new cases (Cancer Research UK, 2017). Women with hormone receptor-positive breast cancer comprise 70% of the total breast cancer population. In order to reduce breast cancer recurrence and mortality, women with this type of cancer are prescribed adjuvant hormone therapy for 10 years following primary cancer treatment (Gnant et al., 2021). Hormone Therapy (HT) [i.e., Selective Estrogen Receptor Modulators (SERMs) (e.g., Tamoxifen) and Aromatase Inhibitors (AI) (e.g., Letrozole, Anastrozole, and Exemestane)] work by inhibiting hormone production or by interfering with hormone receptor signals in order to prevent tumour growth. HT is extremely effective for preventing breast cancer recurrence and reducing breast cancer mortality when taken as prescribed (Shien and Iwata, 2020). Tamoxifen reduces breast cancer recurrence by 40–50% in post-menopausal women and by 30–50% in pre-menopausal women (DeSantis et al., 2019). However, adjuvant AI treatment is significantly more effective in post-menopausal women, resulting in a 22% increase in disease-free survival compared with Tamoxifen (Corona et al., 2019).

Despite these important clinical outcomes, HT discontinuation amongst breast cancer patients is common. Discontinuation, defined as the early ceasing of medical treatments, typically occurs in response to adverse medication side effects or drug-drug interactions (Nixon and Kousgaard, 2016). Approximately 50% of breast cancer patients discontinue HT medication earlier than recommended, resulting in a 20–50% increased risk of mortality (Lambert et al., 2018). Medication non-adherence is also common in this population. Adherence is defined as the rate of compliance to a treatment regarding frequency, timing, and dosage (Wassermann and Rosenberg, 2017). Women taking HT are considered non-adherent when the rate of medication compliance falls below the clinical threshold of 80% (Tinari et al., 2015). HT adherence rates are currently estimated at 79% by the end of year 1, reducing to 56% by the end of year 5 (Moon et al., 2017a). Unintentional non-adherence (e.g., forgetting) is the most common form of non-adherence at approximately 31%, while intentional non-adherence is reported to be around 15% (Kimmick et al., 2015).

Previous studies have identified various factors impacting HT adherence including age, ethnicity, socio-demographics and switching HT medications (e.g., from SERMs to AI). However, the only consistent predictor to emerge from these studies are the adverse effects of the medication itself. Most studies report these adverse effects as an overall side effect profile, which typically includes a combination of cognitive, psychological, gynaecological, musculoskeletal and sleep/fatigue-related symptoms (Moon et al., 2017a; Peddie et al., 2021). However, the usefulness of reporting a side effect profile is questionable since the contribution of specific side effects to HT non-adherence remains poorly understood. Also, the majority of studies in this area utilise quantitative, questionnaire-based methodologies to report this overall side effect profile, where presence/absence of side effects are reported as a single yes/no variable (Lash et al., 2006; Bender et al., 2014; Kyvernitakis et al., 2014). In addition, a lack of consistency in published terminology and data measurement sources (e.g., medical records, clinician checklists, self-report questionnaires) make cross-study comparisons challenging (Lambert et al., 2018). Therefore, by adopting a qualitative approach to the lived experience of taking HT and the challenges associated with managing specific HT side effects, we are better able to understand and identify specific determinants of HT non-adherence that may be modifiable through intervention strategies.

Previous qualitative research has consistently highlighted the negative patient experience of debilitating HT side effects. Joint pain, fatigue, poor sleep and difficulty concentrating significantly impact women’s ability to work, their mental wellbeing and quality of life (QoL). However, Peddie et al. (2021) stated that previous qualitative studies have failed to consider differences between different HT treatment approaches. Clinical observations suggest that different treatments (i.e., Tamoxifen and AI) have different side effect profiles, albeit with some overlap. This study attempts to disentangle differences between the impact of side effects based on treatment type, and whether this had any impact on adherence and persistence.

Interpersonal relationships and patients’ sense of identity is also negatively impacted as a result of menopausal symptoms resulting from HT (Peddie et al., 2021). Despite this, a lack of pre-emptive information around HT side effects and a lack of professional follow-up care are consistently reported. Further research is required to identify women’s unmet symptom management and information needs, and the impact of these on HT adherence. Therefore, this article aims to bridge these gaps in the literature by gaining qualitative insight into the lived experience of taking HT medication and how attempts to manage HT side effects impacts adherence behaviour. This has the potential to inform the development and prioritisation of targetted intervention strategies to promote adherence to HT medication, leading to reduced rates of breast cancer recurrence and mortality.

## Materials and Methods

### Design

To obtain insight into the lived experience of adjuvant HT side effects and its impact on adherence to medication, a qualitative approach was taken. This permits a detailed exploration of the daily experiences and perspectives of breast cancer patients, which previous quantitative studies have failed to capture (May et al., 2021).

### Participants and Recruitment

Twenty-five female participants were recruited via breast cancer clinics at the Beatson West of Scotland Cancer Centre (*n* = 14), and through social media and breast cancer charitable organisations (*n* = 11). Study inclusion criteria included age 16 years or above and a current or previous prescription for adjuvant HT for breast cancer (e.g., Tamoxifen, Anastrozole, Exemestane, and Letrozole). Participants were aged between 31 and 67 years (mean age = 48). All participants were English speaking; 24 self-identified as White-British and 1 as Asian-British. Employment status varied across participants and included full-time employment (61%), part-time employment (13%), retired (22%), and unemployed (4%).

### Data Collection

Recruitment and data collection was conducted between October 2020 and January 2021 and ethical approval was granted by NHS Research Ethics Committee. Willing participants either contacted the researcher team directly, or their details were passed to the researcher team by one of the recruitment sites. Individuals who expressed an interest in participating were emailed a participant information sheet, recruitment leaflet and consent form prior to their online interview. Participants were then contacted by the research team directly in order to answer any outstanding questions, confirm consent and arrange a time for the interview. Further verbal consent was obtained from each participant prior to the interviews commencing.

At the beginning of each interview, participants were reminded of their right to withdraw from the study, ensured of their anonymity, and given an opportunity to ask questions. Due to the COVID-19 pandemic, interviews were conducted via Zoom video calls (cameras on) to allow for face-to-face interaction, and permission was sought to record each interview for later transcription. Interviews lasted approximately 45 min and were conducted by two female interviewers: an experienced qualitative researcher and a postgraduate student with experience of conducting qualitative interviews.

A semi-structured interview schedule was constructed based on previous literature on breast cancer, adjuvant HT medication and adherence behaviour. The research team conducted a pilot study (*n* = 5) to test the interview schedule and the data collection protocol. In response to this pilot study, the order of the interview questions was altered to improve the flow of the interview. Given that the purpose of the pilot study was to assess the appropriateness of the interview tool and protocol for data collection, the data gathered during this preparation phase was not analysed. Due to the exploratory nature of the study, a series of broad open-ended questions were developed, allowing the interviewer to probe further as required. Specifically, the interview schedule included open-ended questions covering (i) experience of HT side effects, (ii) management of HT side effects, and (iii) adherence to HT medication. The schedule acted as a guide and was implemented flexibly to allow participants to elaborate on ideas and explore new concepts that emerged during the interview. At the end of each interview, participants were thanked, and permission was granted to retain their contact details for future studies.

### Data Analysis

Interviews were transcribed by the first author (MI) according to Bailey (2008) guide. Prior to commencement of this transcription process, the level of required detail for each transcription was agreed upon by the research team, including that false starts and repetitions were to be omitted. Participants were each assigned an ID number to ensure anonymity. Thematic analysis was conducted in accordance with the six stages proposed by Braun and Clarke (2006). Three coders (MI, NP, and SA) read over each transcript to become familiar with the data, noting content of interest and generating codes. A table of codes was independently created by each researcher, which included a label and definition for each preliminary theme, increasing coding reliability (Terry et al., 2017). Codes were compared between the three coders and the number of agreements and disagreements were calculated. Disagreements were excluded from the data. Inter-rater reliability was calculated, resulting in a moderate score (*k* = 0.46) according to Landis and Koch (1977). However, it is important to note that this was only based on a small proportion (*n* = 6) of the transcripts, which follows Landis and Koch’s method of using a sub-sample of the population. The codes were organised into master and sub-themes and relevant quotes were selected and analysed in relation to the aims of the study. Theme names were selected to capture unique and essential features of shared meanings across the data set.

## Results

Participants reported experiencing many side effects as a result of HT medication, such as physical pain, mental health impacts and cognitive deficits. The most frequent and severe side effects reported across both types of HT were sleep disturbance (*n* = 25), hot flashes (*n* = 20), and joint pain (*n* = 18). Mental health side effects like anxiety (*n* = 17), depression (*n* = 6), and low mood (*n* = 11) were also common. Anxiety was most prevalent among participants taking AIs. Participants taking SERMs commonly reported depression, cognitive side effects such as brain fog and difficulty concentrating and physical side effects like bloating, fatigue and muscle pain. Table 1 lists all side effects reported and their frequency, split by medication type. Some women reported that they had switched their HT prescription (e.g., from SERMs to AI) during treatment, and the side effects reported by these participants are included in both columns of Table 1.

**TABLE 1:** Frequency of reported HT side effects.

Side effect	Frequency	SERM (Tamoxifen)	Aromatase inhibitors (e.g., Exemestane, Letrozole, Anastrozole)
Sleep disturbance	25	6, 7, 9, 10, 12, 16, 17, 18, 19, 20, 21, 22, 23, 24, 25, 26	2, 3, 4, 5, 8, 9, 10, 11, 13, 14, 15, 21
Hot flashes	20	6, 9, 10, 12, 16, 17, 20, 21, 22, 23, 24, 25	2, 3, 4, 5, 8, 9, 10, 11, 13, 14, 15
Joint pain	18	9, 10, 17, 18, 19, 22, 23, 24, 25, 26	2, 3, 5, 8, 9, 10, 13, 14, 15
Anxiety	17	16, 17, 20, 22, 23, 24, 25	2, 3, 8, 9, 10, 11, 13, 14, 19, 21
Brain fog	16	6, 7, 9, 10, 17, 18, 19, 22, 23, 24, 25, 26	2, 3, 9, 10, 11, 15
Fatigue	16	6, 9, 10, 16, 17, 20, 22, 23, 24, 25, 26	3, 8, 9, 10, 11, 15
Difficulty concentrating	11	9, 10, 12, 18, 19, 20, 22, 24, 26	3, 9, 10, 11
Low mood	11	9, 12, 16, 17, 19, 20, 23, 24, 26	9, 13, 14
Memory loss	11	6, 10	10, 11, 14
Stiffness	10	7, 10, 12, 17, 18, 25, 26	2, 10, 13, 15
Muscle pain	9	6, 17, 18, 19, 22, 24, 25, 26	5
Feeling aged	7	12, 20, 23	2, 11, 13, 15
Headaches	7	6, 9, 10	8, 9, 10, 11, 13, 14
Depression	6	12, 17, 19, 20, 22	11
Nausea	6	6, 10, 18, 23, 25	2
Low energy	5	17, 20, 23, 26	3
Night sweats	5	10, 16, 17, 21	10, 11
Bloating	4	6, 10, 18, 20	5, 10
Hair loss	4	6, 9, 20	9, 11
Personality change	4	12, 16, 20, 26	
Heavy periods	3	10, 19, 20	10
Low self-esteem	3	16, 17, 23	
Weight gain	3	6, 17, 20	
Back pain	2	12	5
Bleeding gums	2	20, 22	
Brittle nails	2		11, 15
Facial hair	2	16, 20	
Osteoporosis	2	25	2
Painful intercourse	2	16, 20	
Skin rash	2	17, 24	
Bone thinning	1		2
Carpal tunnel	1		15
Dizziness	1		2
Excessive thirst	1		4
Excessive vaginal discharge	1	16	
Incontinence	1	20	
Itching	1		2
Teeth grinding	1	17	
Tongue ulcers	1	17	
Upset stomach	1		4
Visual impairment	1		2

### Thematic Analysis

**FIGURE 1:**
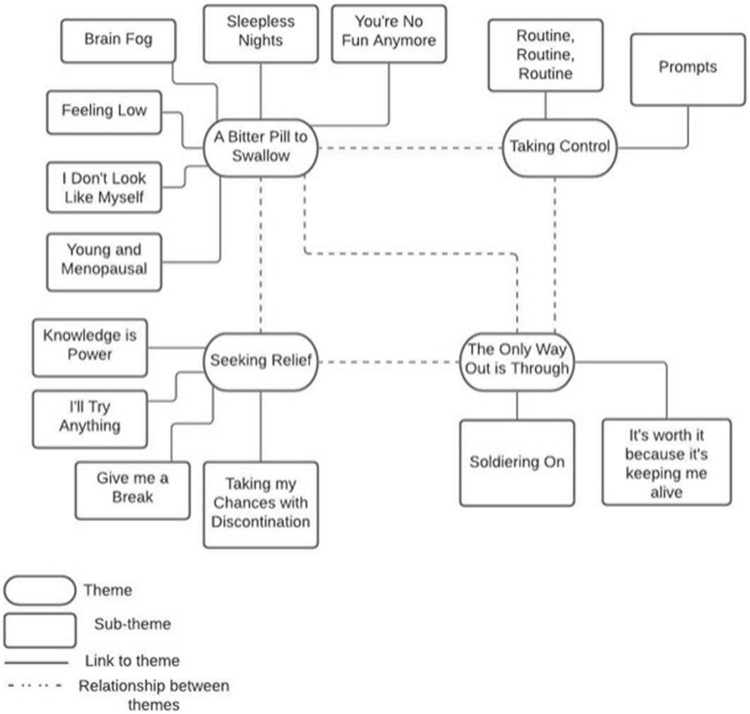
Thematic map demonstrating four main themes and corresponding sub-themes.

The interview data were analysed in order to explore the lived experience of HT and the impact of HT side effects on medication adherence. Four main themes, each with corresponding sub-themes, are presented in Figure 1.

#### Theme 1 – A Bitter Pill to Swallow

The six subthemes within this theme capture the experience and impact of HT side effects and revealed the participants’ daily struggle with several physical, psychological, and cognitive challenges.

##### Sleepless Nights

Sleep disturbance was the most prevalent and troublesome HT side effect reported. Participants attributed their inability to fall asleep and/or remain asleep directly to taking HT. They reported extreme frustration and distress during the night and reported that they felt like their brain had forgotten how to switch off:

*“After 10 days of hardly sleeping, I was banging my head on the wall on the landing, screaming, even during the day. Couldn’t lie down and have a sleep. I don’t know what happens, but it’s a bit like your brain has forgotten how to sleep” (7).*

Sleeplessness impacted participants’ ability to cope well during the day and they became irritable, often snapping at friends and family due to exhaustion. Night-time sleep disturbances and the resulting daytime fatigue negatively impacted their ability to work, as they struggled to wake up early and concentrate throughout the day. Some were even forced to cut down their working hours as a result. Participants felt that good quality sleep was essential for optimal daily functioning and when they did not achieve this, they were discouraged and frequently contemplated HT discontinuation.

##### Brain Fog

Brain fog was described by many participants as a negative HT side effect that impacted daily functioning, memory, and concentration. Participants reported frequently forgetting important information and events. During the interviews, many participants repeatedly lost their train of thought or forgot words mid-sentence. Some participants compared this longer-term HT side effect to “chemo brain,” a well-known cognitive impact of chemotherapy:

*“Honestly, it’s like when you have chemo brain. It’s really funny how things disappear, and you forget about a lot of things, even for the simple thing of my mum bought me pyjamas and I was actually in the shop with her in March. I had no memory of even looking at them. We went to the shops and she bought me them for Christmas…Did I honestly pick them?” (26).*

##### Feeling Low

Participants’ distress and anguish was palpable in the interviews. Many reported negative emotional changes and feeling very low, with some participants describing feeling trapped or not themselves since initiating HT. Others reported feeling anger, distress, and severe mood swings. Emotional changes were reported as serious and never-ending, causing many participants to report that their low mood negatively impacted their motivation to adhere to HT.

*“I’m very moody as well, that’s the other thing. It’s made me really….like my levels of patience are*…. *It’s just, I don’t know. It’s just, imagine you on one of your worst days of the month and it’s like that every single day. That’s kind of how it feels at the moment. It makes it difficult to keep taking the medication” (20).*

##### I Don’t Look Like Myself

Participants reported significant and troubling changes to their physical appearance, like weight gain, bloating, hair thinning, and joint stiffness, as a result of HT. Severe joint stiffness caused them to “feel old” and impacted their mobility:

*“The reason I changed from [Tamoxifen] was because I couldn’t get up off the floor and needed help to walk because of the stiffness” (7).*

These physical changes were upsetting and negatively impacted self-esteem and daily functioning. Participants described their daily battle with these physical changes, and some reported the impact this had on their motivation to persist with their HT treatment. However, many also described their determination to persist with HT, despite its negative physical impacts.

##### You’re No Fun Anymore

Participants’ social lives were negatively impacted by HT side effects. Fatigue, poor sleep, depression, anxiety and joint pain resulted in participants feeling unable to attend social gatherings. Some felt excluded from social groups as they were considered too sick to attend events, and thus, not invited. Furthermore, attending social events was now less enjoyable as they worried about remembering their pills, painkillers, fan, etc., resulting in heightened anxiety:

*“I mean all throughout treatment you feel so crap that you don’t really want to see anybody and then now you know it just it also affects the enjoyment of going out and doing something*…*now I have to think have I got a fan? Have I got paracetamol? Have I got my IBS tablets? In case you start feeling a bit anxiety and it starts to hurt. Have I got my cool mat so that I can actually sleep at night?… Have I got all my different medications? So it takes the enjoyment out of stuff like that” (8).*

Participants often expressed their extreme exhaustion by evening and being “unable to move.” Some participants developed strategies to cope with this such as taking daytime naps or planning afternoon activities instead.

##### Young and Menopausal

A clear distinction between older and younger women’s experience of HT menopausal symptoms emerged from the data. Participants forced into early medical menopause felt practitioners and older women did not understand their concerns. Many were appalled by the lack of concern surrounding the sexual implications of HT, like low libido and painful intercourse. They felt that practitioners were apathetic toward younger women’s menopausal side effects and did not offer adequate solutions. Younger participants reported feeling upset when their healthy counterparts entered menopause at a slower and less severe pace:

*“I’m just like…no sympathy because your [menopause] has been a slow nice decrease… You’ve already had children… very, very different when all your friends are on their third baby…Yeah, they kind of had two babies in the time that I’ve been kind of getting through this” (15).*

A significant consequence of being young and menopausal is the impact on fertility and the ability to have children. Younger participants described this as a hugely distressing aspect of taking HT.

#### Theme 2 – Seeking Relief

The four subthemes within this theme explore participants’ coping strategies for HT side effects, such as seeking information, lifestyle changes as a result of fear of recurrence (FoR), intentional non-adherence and discontinuation.

##### Knowledge Is Power

Participants attempted to take control of HT side effects by conducting their own research before and during treatment. This allowed them to gain information that they had not received at clinic appointments. Online support groups and forums reassured participants that they were not alone. This resulted in them sharing experiences, coping advice and comfort with others, which helped them to remain adherent to HT. However, some women reported their preference to initiate HT unaware of the side effects and only manage them if and when they appeared.

*“I’ve read the pack that comes with it and I have joined a lot of cancer forums. There’s actually a Tamoxifen support group online and so I joined several. I think no, I didn’t know. I didn’t know what to expect and I think what I found is that it’s very different for women” (22).*

##### I’ll Try Anything

Participants were willing to try any intervention that might help relieve their HT side effects including exercise, walking, acupuncture, reflexology, relaxation, and herbal remedies:

*“I went and did an acupuncture course at the McMillan Centre where I went every week for 8 weeks and they did acupuncture on me to help with some of the hormonal side effects and then they gave me acupuncture needles so I could actually do myself at home to help with the hot flushes mainly, which did really help” (11).*

Many participants made dietary changes, avoiding sugary and spicy foods, and limiting alcohol to prevent the exacerbation of hot flashes. Some also limited caffeine intake during the afternoon, to prevent sleep disturbance. All participants made lifestyle changes in order to help them to adhere to their medication.

##### Give Me a Break

Overall, most participants reported reasonably good adherence to HT, but some occasionally became intentionally non-adherent, with the permission of their Oncologist. Taking breaks and altering the dosage to alleviate side effects was common when switching between HT treatments. Participants were surprised that their treatment was not as rigid as they expected it might be and there was scope for some flexibility. Breaks were permitted providing that the total duration of the treatment equates to 10 years.

*“Also, they’re not that bothered about you taking a break. It seems to be your total time that counts. So, what they’ve said is 10 years total treatment. So, you just take off the time for a break so someone stop to have a baby or something. So just add a year onto your treatment kind of plan so that was fine” (10).*

Participants reported consulting with oncology professionals to make a shared decision, instead of simply discontinuing HT.

##### Taking My Chances With Discontinuation

Discontinuation was not common in our study. However, two participants decided it was in their best interests to terminate HT treatment due to their experience of side effects. Prior to discontinuation, there were several attempts made to tailor the treatment to reduce side effects but these did not have sufficient impact. The decision to discontinue was not taken lightly but participants reported losing motivation to continue, despite understanding the potential risks of stopping HT:

*“I stopped on the 16th of August 2019. Then I decided, after my family kept getting on and on and on at me, I thought I’d try again. And so I spoke to the oncologist and I said I would start again on October 2019. But I couldn’t face it. I thought I can’t do it, so I went back to her. And we talked about an oophorectomy and taking Zoladex*… *So, we thought about maybe Zoladex and then trying to do Letrozole. So I said that I would try Letrozole*… *and I had a really hard think about it and I decided just not to take anything in and just decided said to the doctor that I’m just going to take my chances and see what happens” (17).*

During her brief break, participant 17 experienced a sense of normality and could not face re-initiating HT. The presence of multiple, severe, side effects interfered with her QoL and she ultimately chose to spend the remainder of her life living without having to manage them.

#### Theme 3 – Taking Control

The two subthemes within this theme convey the active role participants took to gain control over HT treatment by adopting various strategies to ensure medication adherence.

##### Routine, Routine, Routine

Adopting a routine, such as taking HT at the same time each night, made it easier for participants to manage adverse side effects. Most participants described the trial and error of implementing various routines, until a suitable one was established. This helped them to self-manage the impact of HT, by reducing the experience of certain side effects like nausea and poor sleep. While some preferred administering HT in the morning, others fared better by taking it at night. Participants reported their medication routine as “automatic,” and that this habit played an essential role in promoting HT adherence by minimising its unpleasant impacts:

*“Like I said, I started off by taking it in the morning when I first started it and I felt really nauseas. So I then I thought, I’ll move it, so I moved it to later and then I moved it to later again. So I tend to take it about 8:00 PM, probably 7:00 PM. Yeah, sometimes 7. And I’ve made it. I’ve developed a real habit, you know, like you have to do to remember things, so I have it in the same place, etc. And I’ve rarely forgotten it because I make it a real conscious habit” (25).*

##### Prompts

Participants reported improved adherence to HT by using prompts. These prompts included pill boxes, medical apps, and placing pills in a visible location. This allowed them to self-regulate their adherence and provided a sense of autonomy and control over their treatment.

“Oh, I have a reminder on my phone. Otherwise yes, I use. Gosh, what’s it called? Medi Medi safe maybe? And I use it for everything you did for this. I use it for vitamin tablets. Yeah Medisafe, but otherwise I’m still always surprised when the alarm goes off and I’m like Oh yeah, I didn’t take that. Yeah, it’s like I’ve been taking meds for, it must be four and a half years now. It’s not like it’s new. So, so yeah, I rely on technology to make sure I take my medication” (10).

Self-management, using routine and prompts, was a common strategy that successfully combatted the cognitive impact of HT such as poor memory, and prevented unintentional non-adherence.

#### Theme 4 – The Only Way Out Is Through

The two subthemes within this theme capture participants’ willingness to persist with HT in spite of adverse side effects in order to minimise their risk of recurrence.

##### Soldiering On

Despite the negative impacts of HT, most participants were determined to persist with their treatment in order to beat cancer. Many reported fears of cancer recurrence as their main motivation behind these efforts to remain adherent:

*“I just don’t take the risk in case I get a recurrence, I want to know that I’ve done everything I possibly could to stop it, and I wouldn’t forgive myself if that happened and I’ve been, you know, like taking a break caused that” (22).*

They were motivated to overcome negative side effects and live their lives as they did prior to cancer. Therefore, instead of abandoning treatment, most participants remained committed to managing their experience of adverse side effects by implementing small changes, such as napping during the day and taking regular breaks:

*“And if you then add for you know fatigue caused by lack of sleep. On top of that I just feel rubbish and I find it very difficult to do even you know clean the house be bothered to get out my pyjamas so it really does impact me. So yes, it’s a bit of a battle. I generally just try and sort of forced my way through it and then maybe have a little nap” (11).*

##### A Necessary Evil

Participants reported a sense of inner conflict when deciding whether to persist with HT. Despite being aware of the potentially fatal consequences of discontinuation, they often contemplated early termination of their HT treatment. However, most participants felt HT was worth the struggle and persisted, despite the consequences. The long treatment duration was reported as demoralising, causing participants to question whether its benefits outweighed its consequences:

*“I feel like it’s worth it because it’s these things keeping me alive and I’d rather be alive with the side effects than not. But yes, I mean, my life is completely different and there isn’t really a day where I feel normal in myself. You know where I don’t have some kind of physical side effect and I’ve always been a hugely active and healthy person. So for me it is very strange kind of constantly battling with something or another. But for me, I you know I don’t mind because I feel like it is a necessary evil” (11).*

## Discussion

This qualitative study aimed to explore breast cancer survivors’ lived experience of adjuvant HT and its impact on adherence behaviour. Gaining insight into the lived experience of taking HT and exploring how breast cancer survivors manage HT side effects can help to provide greater understanding of the impacts of the treatment and how adherence might be maximised. Four key themes were identified: “A bitter pill to swallow,” “Seeking relief,” “Taking control,” and “The only way out is through.”

Participants reported an arduous lived experienced of HT, with accounts of multiple, severe and chronic side effects impacting their daily lives. The most frequently reported and severe side effects were sleep disturbances, hot flashes, musculoskeletal pain, and anxiety. These side effects are commonly reported as distressing and interfere with breast cancer survivors’ QoL (Desai et al., 2013; Moon et al., 2017b). Hot flashes are the most frequently reported Tamoxifen side effect (Lorizio et al., 2012). However, we found that sleep disturbance was the most prevalent across Tamoxifen and AIs. Insomnia is an increasingly recognised adverse side effect of both types of HT and our findings are consistent with previous studies investigating HT and insomnia (Lowery-Allison et al., 2018).

Poor sleep adversely affected participants’ ability to work and function productively during the day, due to increased daytime fatigue, poor concentration and memory deficits. These findings are in line with research conducted by Fitch (2020). These cognitive consequences of poor sleep has implications for adherence behaviour, as they may lead to unintentional non-adherence and subsequently, early mortality (Guo et al., 2017; Brownlow et al., 2020). Most participants attributed sleep disturbance to HT as a result of hot flashes and pain, negatively impacting HT adherence (Kwak et al., 2020). However, others reported sleep disturbance as a direct, independent side effect of HT. Some participants attributed their sleep disturbance to anxiety around cancer recurrence, a potential contributing factor for insomnia chronicity (Hall et al., 2019).

Future research should attempt to explore the relationship between these side effects in more detail. Given the high correlation between sleep, anxiety, pain and menopausal symptoms (Toprak and Erden, 2019), it is possible that focussing on one as a key intervention target may have generalised benefits for the others. Sleep is a good place to start. Aside from its prevalence in the breast cancer survivor population, it impacts upon both intentional (“I stopped my hormonal therapy because it disturbed my sleep”) and unintentional (“I am so tired that I can’t remember if I took my hormonal therapy”) non-adherence. Poor sleep is also well managed using Cognitive Behavioural Therapy, an evidenced-based non-pharmacological intervention that is effective and acceptable in the breast cancer population (Espie et al., 2008; Fleming et al., 2010). Importantly, research shows that improving sleep as a target variable has generalised benefit for co-morbid symptoms such as anxiety and pain (Fleming et al., 2014; Luik et al., 2017; Haack et al., 2020). Better understanding of the role of poor sleep in the expression of other common HT side effects is the next step in understanding the complex relationship between these side effects and HT adherence and will help identify key intervention targets to improve HT adherence.

Despite experiencing significant impacts on emotional wellbeing, most participants persisted with HT. However, many did report that they frequently contemplated non-adherence and early discontinuation due to the experience of side effects from the medication. Physical changes such as weight gain and hair loss contribute to negative body image and sexual dysfunction, which is associated with poor emotional wellbeing (Brandão et al., 2017). However, high self-efficacy has been linked with reduced negative physical health and higher emotional wellbeing in this population. Most participants’ adherence to HT increased after implementing coping strategies such as dietary changes, relaxation techniques and seeking information. This behaviour promotes HT adherence by reducing adverse impacts of side effects (Shelby et al., 2014). Many participants also improved HT adherence by establishing a routine using strategies such as medication apps and pillboxes, which seem to be effective in promoting adherence to treatment for chronic illness (Sanders and Van Oss, 2013; Schwartz, 2017; Morawski et al., 2018).

Intentional non-adherence via breaks and altered dosage was occasionally implemented to improve adherence. While this mostly encouraged adherence, participants who discontinued decided to do so after taking such breaks, citing various reasons such as the presence of multiple side effects and a lack of motivation. Therefore, it seems that periods of intentional non-adherence may pose as a risk factor for complete discontinuation – something that should be considered by practitioners when permitting breaks (Brett et al., 2018).

Despite participants reporting significant HT side effects, most claimed to be highly adherent to their HT medication and felt motivated to continue to prevent cancer recurrence. This is inconsistent with previous data that reports high HT non-adherence at 79% (Kimmick et al., 2015; Moon et al., 2017a). However, many of our participants reported fears of cancer recurrence, which may not be representative of the breast cancer survivor population as a whole (Dunn et al., 2015; McGinty et al., 2016). Fear of recurrence, beliefs of efficacy and determination to stay alive for families acted as motivation for HT adherence and persistence. While remaining adherent, participants reported struggling internally, making a daily quantity vs. quality decision. Our findings align with the necessity-concerns framework, which hypothesises that if beliefs about medication necessity outweigh concerns, patients are likely to remain adherent (Horne et al., 1999). Studies have consistently found this pattern where beliefs of efficacy are directly related to adherence, as negative efficacy beliefs are usually followed by non-adherence (Moon et al., 2017a).

In future practice, frequent follow-up appointments should be implemented to monitor HT side effects, provide reassurance of HT efficacy and reduce fears about cancer recurrence. This would provide an opportunity for patients to consult specialists about adverse side effects and efficacy of potential coping strategies. Practitioners should follow a person-centred approach by involving patients in their own treatment and thereby provide a sense of autonomy (Brett et al., 2018).

### Study Limitations

A limitation of this study was the issue of selection bias as we recruited from support services and, therefore, heard less from women who chose to discontinue. This is often a problem in health research (i.e., Yusufov et al., 2021) as it is difficult to recruit people who drop out of interventions of any kind, as they tend to disengage. Therefore, pathways to recruit participants with this experience is more challenging (Kalpakidou et al., 2019). Finally, whilst a relatively large participant sample was recruited, the sample was not ethnically diverse (96% White) and therefore might not reflect the experience of all breast cancer patients (Kihlstrom, 2021).

### Conclusion

Participants described their lived experience of HT as an incessant struggle to manage side effects and consequently, to develop strategies to promote adherence. Adverse side effects from HT, such as sleep disturbance, hot flashes pain and anxiety, impair QoL and increase the likelihood of participants becoming non-adherent to their medication. Poor sleep specifically has consequences for unintentional non-adherence via its impact on memory and concentration. In order to maximise HT adherence in breast cancer survivors, the lived experience of HT should be monitored in order to identify individuals who may benefit from specific, targetted intervention strategies aimed at alleviating these side effects. Evidence-based interventions for the most common HT side effects (sleep disturbance, hot flashes, musculoskeletal pain, and anxiety) may be a supportive and effective way of promoting HT adherence, which is key to reducing breast cancer mortality rates. Future work should focus on the identification and implementation of evidence-based interventions targetting the most commonly reported HT side effects in order to promote HT adherence and quality of life for breast cancer survivors.

## Data Availability Statement

The raw data supporting the conclusions of this article will be made available by request to the authors, without undue reservation.

## Ethics Statement

The studies involving human participants were reviewed and approved by the Yorkshire and the Humber – Leeds West Research Ethics Committee. The patients/participants provided their written informed consent to participate in this study.

## Author Contributions

MI and NP conducted the interviews with participants and led the data analysis process, assisted by SA and AD. All authors were involved in the writing, editing, and reviewing of the manuscript.

## Conflict of Interest

The authors declare that the research was conducted in the absence of any commercial or financial relationships that could be construed as a potential conflict of interest.

## Publisher’s Note

All claims expressed in this article are solely those of the authors and do not necessarily represent those of their affiliated organizations, or those of the publisher, the editors and the reviewers. Any product that may be evaluated in this article, or claim that may be made by its manufacturer, is not guaranteed or endorsed by the publisher.
